# Endovascular treatment of massive hemorrhage arising from inferior thyroid artery after fine needle aspiration of thyroid: a case report

**DOI:** 10.1186/s12893-021-01184-5

**Published:** 2021-04-27

**Authors:** Ho Sig Jang, Yook Kim

**Affiliations:** Department of Radiology, Chungbuk National University Hospital, 776, 1sunhwan-ro, Seowon-g, Cheongju-si, Chungcheongbuk-do Republic of Korea

**Keywords:** Fine needle aspiration, Thyroid gland, Hemorrhage, Transcatheter arterial embolization, Inferior thyroid artery

## Abstract

**Background:**

Fine needle aspiration (FNA) of the thyroid gland is an effective and safe method for evaluating thyroid nodules; catastrophic complications following FNA of thyroid are rare. Massive hematomas with active bleeding leading to airway compromise are extremely rare complications of FNA, with only a few reported cases in literature.

**Case presentation:**

An 80 year-old man presented to the emergency room with severe respiratory distress, four hours after undergoing thyroid FNA for the evaluation of a thyroid nodule. An axial neck computed tomography (CT) revealed a large hematoma in the retropharyngeal space that caused anterior deviation of the trachea, with extravasation of contrast media suggesting active bleeding within the hematoma. Right subclavian angiography identified active bleeding from the right inferior thyroid artery (ITA). Transcatheter arterial embolization (TAE) was successfully performed with *n*-Butyl cyanoacrylate (NBCA). Follow-up CT done three weeks after the procedure revealed a low-density lesion in the retropharyngeal space, indicating an old hematoma. The patient recovered well and was discharged 6 weeks later.

**Conclusion:**

Massive hemorrhage arising from the ITA is a rare but possible complication following FNA, which can potentially be fatal. Appropriate preventive measures should be taken while performing FNA, especially in patients on long-term anticoagulant drugs, and prompt intervention is mandatory for patients with acute hematoma after FNA.

## Background

Fine needle aspiration (FNA) of thyroid lesions is a common technique widely used for the diagnostic workup of thyroid nodules [1–4]. It has proven to be a safe, reliable, and effective tool over the years. Except for mild neck discomfort, complications related to this procedure are few; massive neck hematomas after FNA are a rare event, and several cases have been reported in the literature [5–7]. Furthermore, injuries of smaller vessels, like the inferior thyroid artery (ITA), are extremely rare. Despite being uncommon, this complication has the potential to be highly lethal, and several authors have recommended aggressive management [6, 7]. Surgical ligation and/or excision and transcatheter arterial embolization (TAE) are possible treatments for this rare condition [3–6]. Herein, we present the case of an elderly patient who experienced acute respiratory distress due to a large cervical hematoma in the retropharyngeal space following FNA. To date, this is the first report of a massive hemorrhage arising from the ITA after FNA of thyroid, successfully controlled with TAE using *n*-Butyl cyanoacrylate (NBCA).

## Case presentation

**Fig. 1:**
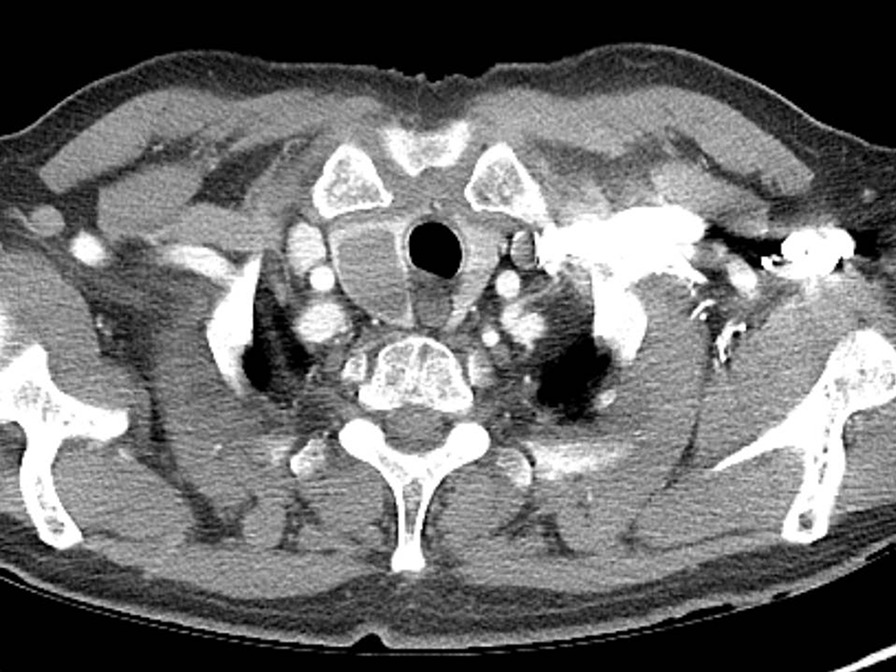
Contrast-enhanced chest computed tomography (CT) taken before ultrasound-guided fine needle aspiration (FNA) of the thyroid gland reveals well-defined low density nodule in right lobe of thyroid gland

**Fig. 2:**
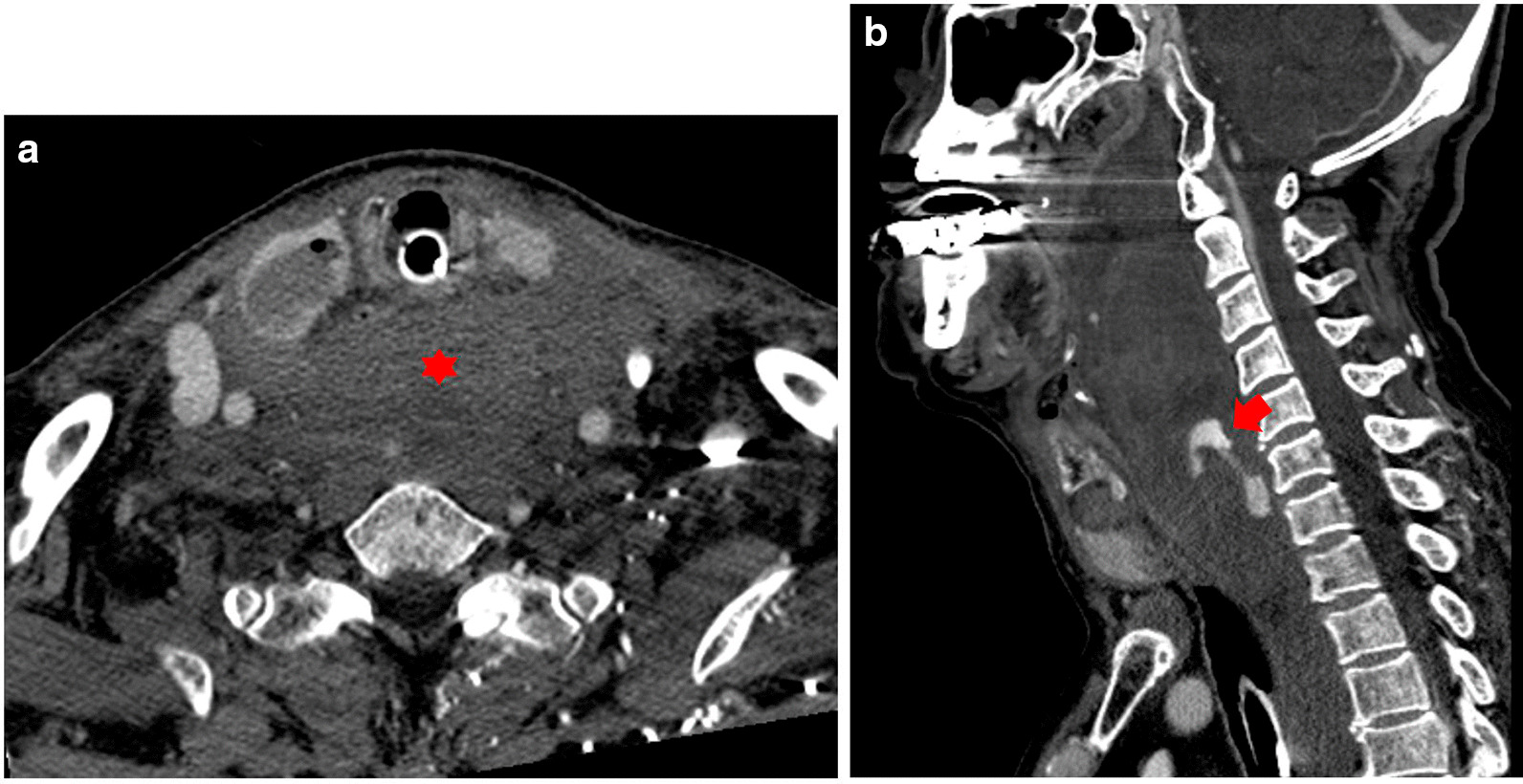
**a** An axial neck CT take after thyroid FNA underwent on emergency department reveals a large hematoma (red asterisk) in the anterior neck space with anterior tracheal deviation and nodule in the right thyroid lobe with intra- and extra nodule air-bubbles caused by the fine needle aspiration. b Sagittal CT scan shows extravasation of contrast media suggesting active bleeding (red arrow) within the hematoma

**Fig. 3:**
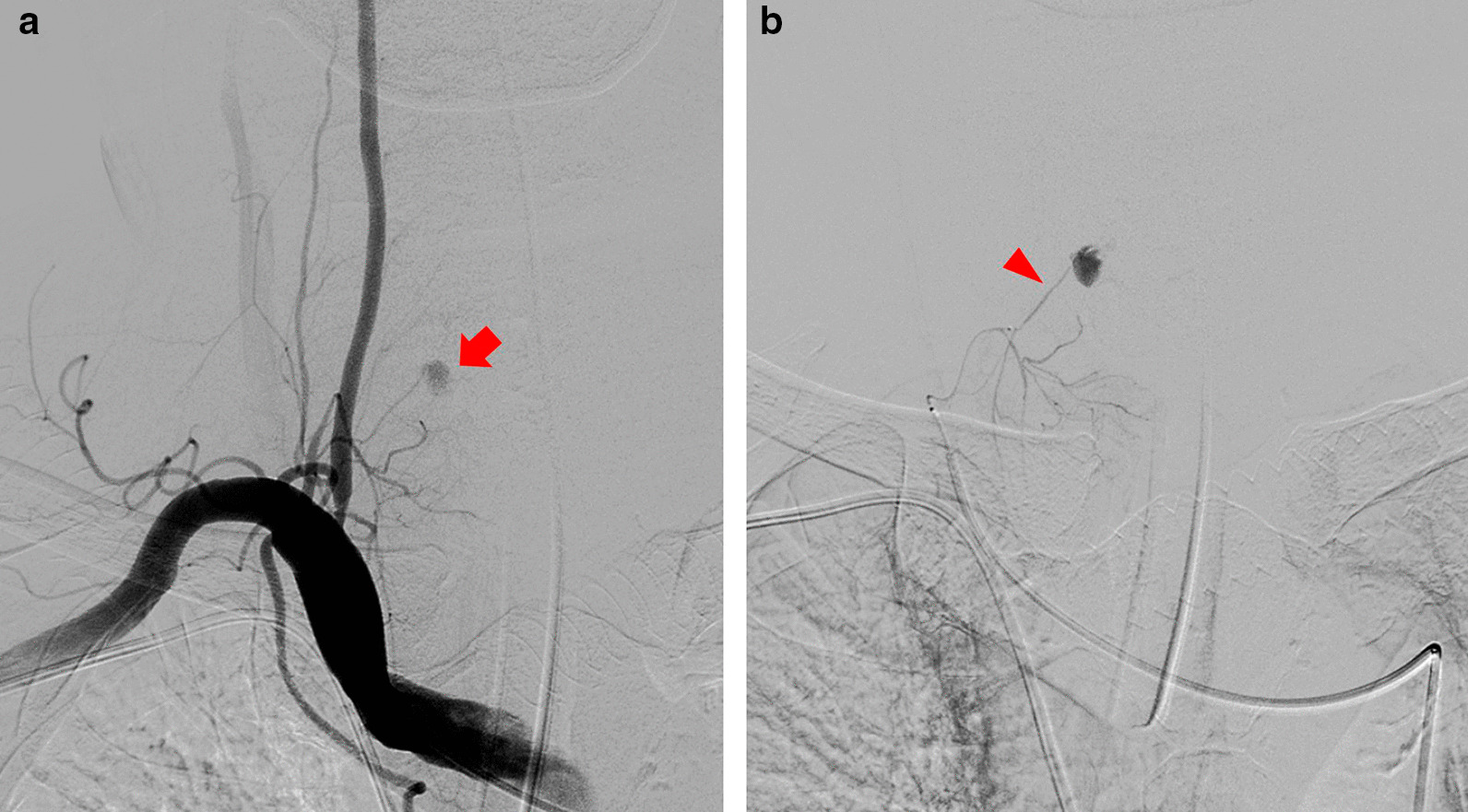
**a** Right subclavian angiography shows active bleeding (red arrow) in the neck, corresponding to the CT image. b Right thyrocervical trunk is selected and the culprit branch is identified as the right inferior thyroid artery (red arrow head)

**Fig. 4:**
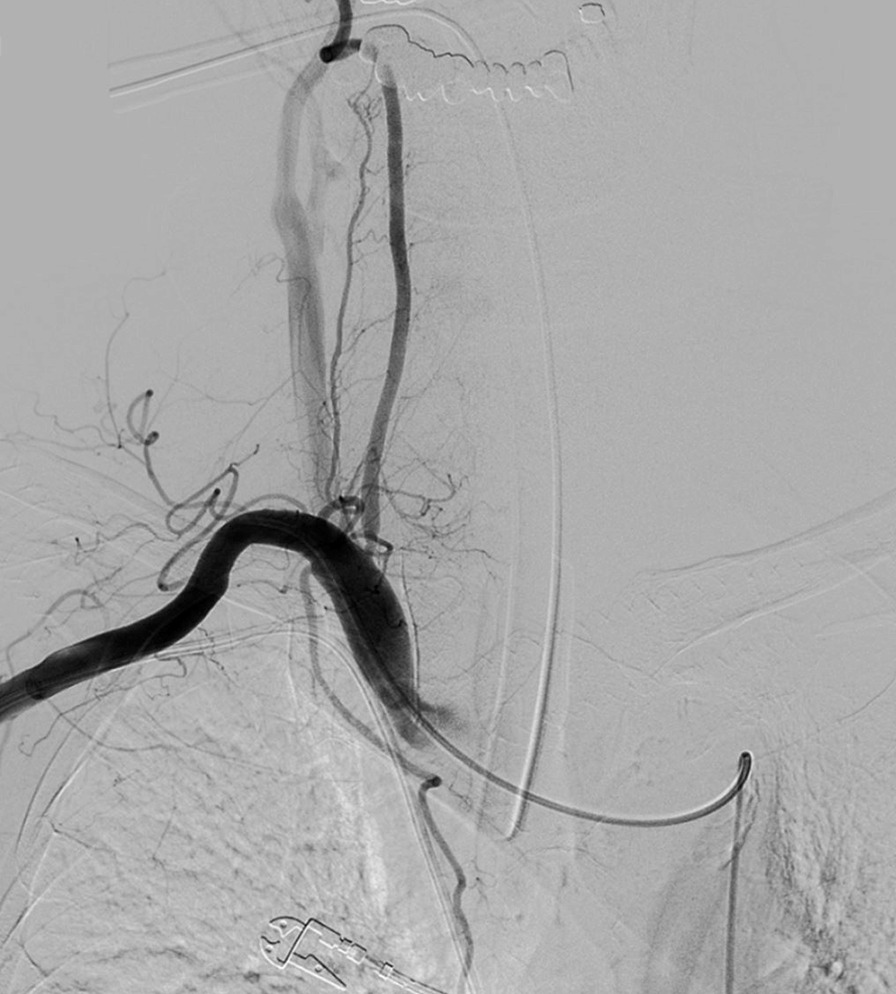
After superselection of the right ITA using microcatheter, embolization of the right ITA is performed using NBCA, and a post-embolization angiogram reveals successful hemostasis without active bleeding

**Fig. 5:**
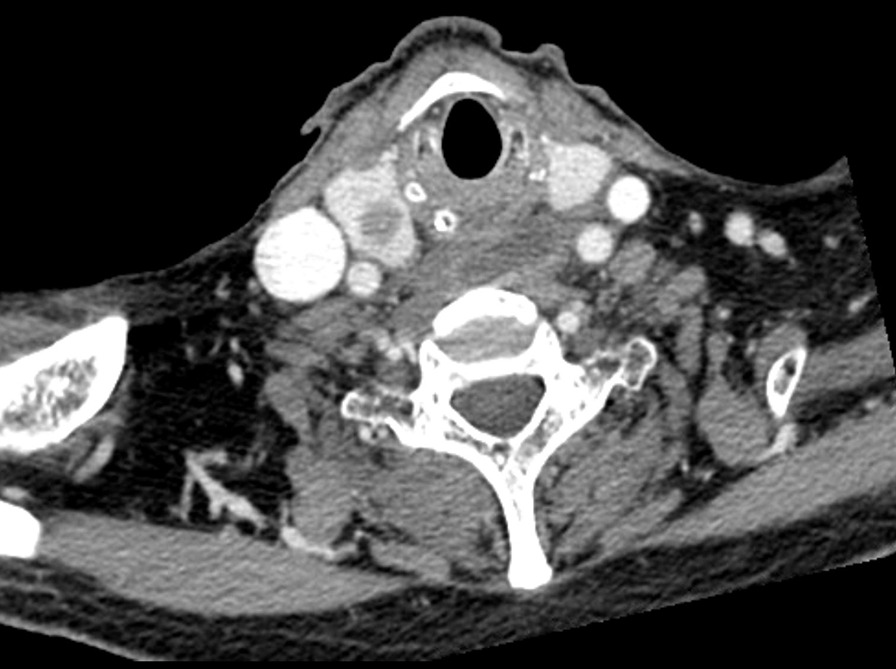
Follow-up CT taken three weeks after transcatheter arterial embolization reveals a low-density lesion indicating an old hematoma in the posterior neck space

An 80 year-old man underwent ultrasound-guided FNA of the thyroid gland with a 23-gauge needle for the evaluation of a 1.8 × 2.2 cm well-defined, cystic nodule with internal tiny solid portion in the right lobe, which was identified by contrast-enhanced chest computed tomography (CT) taken one month before FNA (Fig. 1). Two passes were made into the lesion during the procedure. There were no complications during the procedure. Three hours later, the patient experienced swelling and progressive pain in the anterior neck. He was referred to the emergency room of our hospital with severe respiratory distress, four hours after the FNA. Emergency intubation was performed, and the patient remained hemodynamically stable. The patient was on a long-term anticoagulant medication prescribed for cardiovascular disease. Although the platelet count and liver functions were within normal range, the International Normalized Ratio (INR) was 1.35 (normal range: 0.085–1.15). An axial neck CT taken on emergency department revealed a large hematoma in the retropharyngeal space that caused anterior tracheal deviation (Fig. 2a). The sagittal neck CT showed extravasation of contrast media, suggesting active bleeding within the hematoma (Fig. 2b). Angiography was performed as a primary diagnostic and therapeutic procedure. Right subclavian angiography showed active bleeding arising from the right thyrocervical trunk in the neck, corresponding to the CT image (Fig. 3a). The right thyrocervical trunk was selected, and the culprit vessel was identified as the branch of the right inferior thyroid artery (ITA) (Fig. 3b). After superselection of the right ITA using microcatheter, embolization of the right ITA was performed using NBCA, and a post-embolization angiogram revealed successful hemostasis without active bleeding (Fig. 4). Although the retropharyngeal hematoma still remained, the patient’s respiratory rate was very stable under intubation state, and there was no evidence of increase in hematoma, which was confirmed on a daily follow up neck x-ray. Considering the risk of procedure-related complications, we decided to maintain conservative management for the hematoma. Six days after the embolization, when the patient was able to breathe around the endotracheal tube, it was removed without accident. Follow-up CT taken three weeks after TAE revealed a low-density lesion in the retropharyngeal space, indicating an old hematoma, which was smaller than it was before TAE without further treatment such as percutaneous drainage or open excision (Fig. 5). The patient recovered well and was discharged four weeks later. The hematoma in the retropharyngeal space was completely eliminated, which was confirmed by follow-up CT taken three month after the TAE, performed in an outpatient clinic. Unfortunately, we failed to obtain enough blood sample through the FNA, so failed to obtain the final pathological report. Additional FNA was not performed because the patient refused to take further work up for the thyroid nodule.

## Discussion and conclusions

Fine needle aspiration is an effective and proven routine procedure for the differential diagnosis of thyroid nodules, with a high sensitivity and specificity [1–3]. The incidence of major and minor complications after FNA of thyroid is very low. The most common complication is hematoma formation which is usually asymptomatic and resolves completely [2]. Massive hemorrhage after FNA of thyroid is extremely rare. Several reported cases have described the formation of a retropharyngeal hematoma large enough to become life-threatening. These developed over a few hours with the patient initially presenting with hoarseness and mild dysphasia and rapidly progressed to severe respiratory distress [4–6]. The differential can be complex in these patients, since the symptoms overlap with those of allergic reactions, stroke, or an infectious etiology [1]. Therefore, when lethal bleeding is suspected after FNA, diagnostic imaging should be performed immediately to facilitate immediate interventions. CT is helpful in identifying the origin of bleeding because the multidetector CT, now commonly used, provides better image quality with rapid image acquisition and various multiplanar image reformations. CT may also help the interventional radiologist to plan for the procedure and thereby decrease the number of angiograms required to localize the bleeding site. In our patient, the hematoma was located at the posterior aspect of the thyroid gland on CT. Therefore, injury to the posterior branch of thyroid artery was suspected to be the bleeding focus and also revealed the active bleeding in the retropharyngeal space, the hemorrhage site suggesting that ITA was the culprit. Thus, the bleeding vessel was determined before angiography.

The thyroid gland has a rich blood supply, derived from the superior and inferior thyroid arteries bilaterally. The superior thyroid artery (STA) branches out from the external carotid artery just below the level of the greater cornu of the hyoid bone and primarily supplies the upper and anterior part of the thyroid gland [7]. The inferior thyroid artery (ITA) arises from the thyrocervical trunk and supplies the posteroinferior parts of the gland. High-resolution ultrasounds as well as color Doppler study can visualize these vessels on examination of the neck. During interventional procedures, careful mapping of these vessels in the vicinity of the concerned lesion before biopsy is significant, to trace a safe path for the needle and avoid iatrogenic complications such as hematoma and arterial rupture. Arterial injuries during FNA are usually caused by inserting the FNA needle in the upper and anterior part of the gland. As a result, cases concerning STA injury during FNA of thyroid have been reported more than that of ITA [4, 8]. Conversely, ITA injury is more common during catheterization via the right internal jugular vein, considering that the proximal portion of ITA has an ascending course just lateral to the internal jugular vein [8, 9]. To date, this is the first reported case dealing with ITA rupture caused by FNA of thyroid. We postulate that the massive hematoma formation during FNA in this case could be caused by the following factors. First, the aspiration needle was probably aimed too low and deep in the neck leading to the ITA injury. Second, even though ultrasound-guided direct compression was applied to the needle entry on the skin, this may not have been effective due to the deep location of the concerned anatomical structures. Third, an arterial puncture resulting in a periarterial hematoma is usually self-limiting with the application of pressure. However, if the arterial injury is severe or if there are pre-existing coagulation abnormalities, continuous arterial bleeding can occur [4]. The INR was slightly prolonged in this patient prior to the FNA; the patient was on long-term anticoagulant drugs for cardiovascular disease. Therefore, prior to FNA of the thyroid gland, careful assessment of the patient’s medication history of antithrombotic and anticoagulant drugs is necessary to determine an adequate period of withdrawal of the offending drug.

Treatment options for hemorrhage from ITA include ultrasound-guided compression (UGC), surgical repair, and transcatheter arterial embolization (TAE) [1–3]. UGC therapy induces spontaneous within feeding vessels by transiently occluding blood flow. However, obesity, concomitant anticoagulation therapy, and the presence of a large hematoma, especially those located in the posterior neck space, are factors associated with UGC failure [2, 3]. Compared to STA, hematoma due to ITA rupture occupies the retropharyngeal space. Sudden deaths could result from hypovolemia due to excessive blood loss into soft tissues or the thorax or due to airway compression by the hematoma itself, leading to respiratory distress. Therefore, arterial rupture of ITA should be considered emergent and requires aggressive management. Surgical ligation and/or excision is most suitable in these cases, especially with rupture, since airway maintenance becomes troublesome due to the mass effect of the hematoma [10, 11]. However, surgery has the disadvantage of certain risks including recurrent or phrenic nerve injury and wound site infection. Compared to surgical procedures, TAE is a safe and effective treatment option with lower procedure-related complication rates, avoidance of surgical risks, and shorter hospitalization [3]. The type of embolic agent and the methods of delivery of the agent should be dictated based on angiographic findings of vessel injury [11]. Coils are the ideal embolic agent for arterial rupture caused by iatrogenic injury, as they allow precise embolization [11–14]. However, delivering the coil to the bleeding site is not always possible due to small vessel size or tortuous anatomy. Other options include exclusion of the fistula connection with NBCA. NBCA is also widely used for controlling active bleeding following trauma [15–17]. It rapidly polymerizes with blood; therefore, it is beneficial for massive hemorrhages that require urgent hemostasis. Moreover, its capacity for high penetration owing to its liquid nature can help to obliterate small distal vessels. TAE was performed using NBCA in this case; successful embolization was achieved, and the patient was discharged without any recurrent bleeding.

In conclusion, although FNA of the thyroid gland is a useful and safe procedure, inferior thyroid artery injury is an extremely rare complication. Those who perform this procedure should be aware of this possibility to avoid misdiagnosis and lethal outcomes, especially in patients on anticoagulant drugs. TAE is the treatment of choice, safe and effective in patients with massive bleeding caused by thyroid arterial injury.

## Data Availability

The pertaining data are available from the corresponding author on reasonable request.

## Abbreviations

CT: Computed tomography
FNA: Fine needle aspiration
ITA: Inferior thyroid artery
INR: International normalized ratio
NBCA: *n*-Butyl cyanoacrylate
STA: Superior thyroid artery
TAE: Transcatheter arterial embolization
UGC: Ultrasound-guided compression
